# Predicting malnutrition from longitudinal patient trajectories with deep learning

**DOI:** 10.1371/journal.pone.0271487

**Published:** 2022-07-28

**Authors:** Boyang Tom Jin, Mi Hyun Choi, Meagan F. Moyer, David A. Kim

**Affiliations:** 1 Department of Computer Science, Stanford University, Stanford, California, United States of America; 2 Department of Bioengineering, Stanford University, Stanford, California, United States of America; 3 Department of Digital Health Care Integration, Stanford Health Care, Stanford, California, United States of America; 4 Department of Emergency Medicine, Stanford University, Stanford, California, United States of America; Sreenidhi Institute of Science and Technology, INDIA

## Abstract

Malnutrition is common, morbid, and often correctable, but subject to missed and delayed diagnosis. Better screening and prediction could improve clinical, functional, and economic outcomes. This study aimed to assess the predictability of malnutrition from longitudinal patient records, and the external generalizability of a predictive model. Predictive models were developed and validated on statewide emergency department (ED) and hospital admission databases for California, Florida and New York, including visits from October 1, 2015 to December 31, 2018. Visit features included patient demographics, diagnosis codes, and procedure categories. Models included long short-term memory (LSTM) recurrent neural networks trained on longitudinal trajectories, and gradient-boosted tree and logistic regression models trained on cross-sectional patient data. The dataset used for model training and internal validation (California and Florida) included 62,811 patient trajectories (266,951 visits). Test sets included 63,997 (California), 63,112 (Florida), and 62,472 (New York) trajectories, such that each cohort’s composition was proportional to the prevalence of malnutrition in that state. Trajectories contained seven patient characteristics and up to 2,008 diagnosis categories. Area under the receiver-operating characteristic (AUROC) and precision-recall curves (AUPRC) were used to characterize prediction of first malnutrition diagnoses in the test sets. Data analysis was performed from September 2020 to May 2021. Between 4.0% (New York) and 6.2% (California) of patients received malnutrition diagnoses. The longitudinal LSTM model produced the most accurate predictions of malnutrition, with comparable predictive performance in California (AUROC 0.854, AUPRC 0.258), Florida (AUROC 0.869, AUPRC 0.234), and New York (AUROC 0.869, AUPRC 0.190). Deep learning models can reliably predict malnutrition from existing longitudinal patient records, with better predictive performance and lower data-collection requirements than existing instruments. This approach may facilitate early nutritional intervention via automated screening at the point of care.

## Introduction

Malnutrition, in which inadequate or imbalanced nutritional availability leads to deleterious changes in body composition and function [1], may develop as a result of deficient intake, inflammation, hypermetabolism, or malabsorption [2]. 15–60% of hospitalized patients are estimated to be malnourished [3], but the diagnosis is often missed or delayed, leading to preventable adverse outcomes [4].

Many hospital accrediting agencies, such as the Joint Commission, mandate screening of all hospital patients for malnutrition [5]. Most hospitals rely on questionnaires such as the Nutritional Risk Screening 2002 (NRS-2002) [6] or the Malnutrition Universal Screening Tool (MUST) [7], but no universal standard exists [8,9]. These questionnaires commonly measure recent weight loss, body mass index, acute disease effects, and dietary intake. Screening questions are susceptible to subjective interpretation [9], and fail to identify many cases of malnutrition [10]. Therefore, malnutrition is often under-identified or undocumented.

Early detection and treatment of malnutrition has a major impact on clinical, functional, and economic outcomes [10]. Compared to non-malnourished patients, malnourished patients experience hospital admissions of twice the duration and cost. Malnourished inpatients have up to 4.7 times the mortality of the general inpatient population [11]. A malnourished patient is 1.6 times more likely to be readmitted within 30 days of discharge than a non-malnourished patient, and these readmitted patients are twice as likely to be diagnosed with a serious infection, compared to readmitted non-malnourished patients [11].

Critically, malnutrition, once identified, can be treated with evidence-based and relatively low-cost nutritional interventions [12]. Accurate identification and documentation of malnutrition is also necessary for coding, reimbursement, risk-adjustment, and overall accuracy of patient data [13]. In this way, early and accurate identification of malnutrition can improve patient and health system outcomes [5], raising the need for universal and accurate screening.

Despite the importance of early detection, existing methods to screen for malnutrition have substantial limitations. One common screening instrument, MUST, achieved an area under the receiver-operating characteristic curve (AUROC) of 0.662 in one recent evaluation [14]. Recent attempts to improve the detection or prediction of malnutrition have focused on the evaluation of diagnostic criteria, or the optimization of information flow within the hospital [15–18]. Two recent studies have applied random forests, a type of ensemble machine learning model [19], to the prediction of malnutrition. MUST-Plus, a random forests model relying heavily on anthropometric and laboratory values, achieved an AUROC of 0.835 for the detection of malnutrition in one tertiary health center [14]. Talukder and Ahammed applied several conventional machine learning algorithms to the prediction of malnutrition in a high-prevalence pediatric cohort in Bangladesh. Though this study did not report AUROC, their best-performing model was a random forests model trained on demographic and anthropometric information, achieving an overall accuracy of 68.51% [20]. Notably, both of these studies used conventional, cross-sectional machine learning models (random forests), depended heavily on laboratory or anthropometric measurements not collected in all health care encounters, and failed to demonstrate external generalizability.

The present study reports the development and external validation of deep learning models for the prediction of malnutrition from longitudinal patient records. To maximize potential applicability in screening, these models use only passively collected demographic, diagnostic, and procedural information, and the evolution of these features over time, to identify patients who will receive a first diagnosis of malnutrition. To our knowledge, this is the first study to predict malnutrition with demographic and diagnostic information alone, without requiring laboratory or anthropometric results, and to specifically characterize the time interval between prediction of malnutrition and actual diagnosis, during which a focused nutritional assessment and intervention could be delivered.

## Materials and methods

### Data sources and transformations

The study data consisted of comprehensive, statewide administrative databases of emergency department (ED) visits and hospital admissions for California (California Department of Health Care Access and Information), Florida, and New York (Healthcare Cost and Utilization Project). These data sources were selected because they represent large, diverse states, allow tracking of individual patient trajectories across time, and by virtue of their comprehensive, all-payer nature, reduce the risk of results biased to a specific population (such as a specific hospital system or commercial insurer). The study data included 43M Emergency Department (ED) visits and hospital admissions, representing the trajectories of 5.9M unique adult patients in California (CA), Florida (FL), and New York (NY), who had at least four visits between October 1, 2015, when the 10th International Statistical Classification of Diseases and Related Health Problems (ICD-10) diagnostic coding went into effect, and December 31, 2018. Each de-identified visit record included patient demographics (age, sex, race, state income quartile of patient’s zip code), visit characteristics (primary payer, length of stay, number of days since last visit), up to 24 ICD-10 diagnosis codes (truncated to the first three characters), and up to 20 procedure codes (ICD-10 for admissions, CPT-4 for ED visits), which were standardized to Clinical Classification Software (CCS) procedure categories [21]. Patients with inconsistent ages were excluded.

Patients were classified as malnourished upon receipt of one or more diagnoses of protein-calorie malnutrition or cachexia (S1 Table), selected based on the 2016 HCUP report on malnutrition, and reviewed by a Stanford Health Care registered dietitian [11]. For malnourished patients, trajectories consisted of 3–5 visits preceding the first diagnosis of malnutrition. The visit during which malnutrition was diagnosed was excluded, with the goal of developing models to facilitate early identification of malnutrition. Control trajectories consisted of ED visits and admissions without a malnutrition diagnosis during the study period. As for case trajectories, the last visit was excluded, and trajectories consisted of 3–5 visits.

### Training, validation, and test sets

CA and FL trajectories were combined to produce training (51,710 patients; 219,796 visits) and internal validation datasets (11,101 patients; 47,155 visits) with similar numbers of malnourished (39.6%) patients.

To simulate the effectiveness of models as clinical screening instruments, test sets were produced for each state, randomly sampling malnourished and non-malnourished patient trajectories in proportion to the prevalence of malnourished patients in the complete statewide datasets: CA (6.2%), FL (4.9%), and NY (4.0%). The size of the test sets (CA = 63,997, FL = 63,122, NY = 62,472 trajectories) were selected for computational tractability. Although models were trained on data from CA and FL, none of the test sets contained patients used in training or validation (S1 Fig).

### Diagnosis and procedure code representations

In the primary models, diagnoses, procedures, sex, race, income quartile, and payer were represented using one-hot encoding (binary indicators). Age, days since last visit, and admission length-of-stay were normalized to the range [0,1]. Each visit was thus represented as a 2029-dimensional vector (S2 Fig).

Auxiliary models used pre-trained embeddings developed based on coincident diagnosis and procedure codes (S1 Methods). Each code in each patient visit was represented by an embedding vector (S3 Fig), which was averaged to produce a dense embedding in place of the one-hot encodings (S4 Fig). Each visit could then be represented using a smaller, dense vector of length 53–277 (32–256 length embeddings + 21 one-hot encoded demographic features), depending on the size of the embedding used.

### Predictive models

Classifiers were trained to predict whether a patient would receive a malnutrition diagnosis. The demographic baseline models consisted of logistic regression, random forest (n_estimators = 100, max_depth = None) [22], and gradient-boosted tree ensemble classifiers (XGBoost: n_estimators = 100, max_depth = 3) [23], trained on the demographic variables from the last visit of each patient trajectory. An XGBoost model was also trained using all codes from all visits in the trajectory (i.e., “flattened” codes, with temporal and sequence information discarded).

Deep learning models were developed using the long short-term memory (LSTM) architecture [24], a type of recurrent neural network. The LSTM architecture was selected for its performance on time-series data, and demonstrated utility for medical trajectories [25,26]. In this application, LSTMs are used to model the temporal relationships between visits. LSTM models consisted of a bidirectional LSTM layer with 32 units, followed by two fully connected layers, the first with 128 units and a dropout rate of 0.2, and the second with a binary outcome (malnourished or non-malnourished). Binary cross-entropy loss and the Adam optimizer were used with a learning rate of 0.001. LSTM models were developed on both one-hot encoded and embedded visit representations. A cross-sectional model was also trained, without an LSTM layer, on the last visit in each trajectory, with the same hyperparameters except for the learning rate, which was set to 0.0001.

Model predictions were interpreted using model-agnostic Shapley values, which reflect the marginal contribution of each variable in each patient’s trajectory to the model’s prediction [27].

### Stratified analyses, ablation studies, lead time experiments

In order to understand the performance of the models in specific populations, the test datasets were stratified on demographic features. For instance, the test patient trajectories were split into elderly (≥65 years) and non-elderly cohorts (<65 years), and predicted malnutrition for each of these cohorts using the same model.

In order to understand the relative importance of different features, beyond the Shapley values described above, ablation studies were conducted, retraining the same models but excluding a specific feature category, such as all diagnosis codes, for each retrained model.

Prediction *lead time* refers to the number of days between the last visit observed for each patient (i.e., the point of prediction), and the visit containing the first malnutrition diagnosis. For instance, to assess model performance in predicting malnutrition with at least 30 days lead time, any visits occurring less than 30 days before the malnutrition diagnosis were excluded from trajectories. To control for the reduction in trajectory lengths with increasing lead time, performance with trajectories restricted to exactly five visits, after lead time filtering, was also evaluated.

### Statistical analysis of model results

Model performance was characterized using area under the receiver-operating characteristic curve (AUROC), reflecting the relationship between sensitivity and specificity, and area under the precision-recall curve (AUPRC), reflecting the relationship between positive predictive value (PPV, ‘precision’) and sensitivity (‘recall’), which may better reflect the prediction of rare outcomes than AUROC [28]. The 95% confidence intervals (CI) of AUROC and AUPRC were estimated using the Wilson Score interval method [29]. CIs were validated for the best-performing models with bootstrap sampling.

## Results

### Patient characteristics

Malnourished patients had similar characteristics in all states (S2 Table). Among admitted patients, malnourished patients had longer hospital stays, with median length-of-stay between 6 (CA) and 8 (NY) days, compared to 3 (CA, FL) or 4 (NY) days for non-malnourished patients. Malnourished patients were older than non-malnourished patients by a median of 20 (CA) to 23 years (FL, NY). Malnourished patients had more frequent visits, with median intervals between visits ranging from 24 (CA) to 28 (NY) days, compared to 57 (NY) to 77 (CA) days for non-malnourished patients. Women were diagnosed with malnutrition at higher rates than men. By primary payer, Medicare was associated with the highest rate of malnourished patients, consistent with the advanced age of these patients. Among racial/ethnic groups, Asians had the highest prevalence of malnutrition, and Hispanics had the lowest. The proportion of patients with malnutrition diagnoses was highest for zip codes in the highest (FL, NY) and second highest (CA) income quartiles. Tables 1 and S2 show patient characteristics of the subsets and the full datasets, respectively.

**Table 1:** Patient characteristics in test sets used to assess predictive performance.

	California			Florida			New York		
	Total	Malnourished	Control	Total	Malnourished	Control	Total	Malnourished	Control
Sample size	63997	3997	60000	63122	3122	60000	62472	2472	60000
Length of stay, median (IQR), days	3 (2–6)	6 (3–11)	3 (2–6)	3 (2–6)	7 (4–13)	3 (2–5)	4 (2–8)	8 (4–15)	4 (2–7)
Age, median (IQR), years	52 (33–69)	70 (58–82)	50 (32–67)	50 (32–69)	70 (58–82)	48 (32–68)	49 (32–67)	71 (58–82)	48 (31–66)
Time from last visit, median (IQR), days	72 (15–199)	24 (7–75)	77 (16–207)	73 (18–198)	27 (11–77)	77 (18–204)	55 (14–146)	27 (11–68)	57 (14–150)
Income, n (%)									
Unknown	2527 (3.949)	158 (3.953)	2369 (3.948)	2169 (3.436)	114 (3.652)	2055 (3.425)	834 (1.335)	37 (1.497)	797 (1.328)
Quartile 1	13635 (21.306)	815 (20.390)	12820 (21.367)	17472 (27.680)	800 (25.625)	16672 (27.787)	26548 (42.496)	816 (33.010)	25732 (42.887)
Quartile 2	18903 (29.537)	1077 (26.945)	17826 (29.710)	15207 (24.091)	725 (23.222)	14482 (24.137)	10527 (16.851)	387 (15.655)	10140 (16.900)
Quartile 3	16815 (26.275)	1055 (26.395)	15760 (26.267)	17668 (27.990)	838 (26.842)	16830 (28.050)	11504 (18.415)	476 (19.256)	11028 (18.380)
Quartile 4	12117 (18.934)	892 (22.317)	11225 (18.708)	10606 (16.802)	645 (20.606)	9961 (16.602)	13059 (20.904)	756 (30.583)	12303 (20.505)
Payer, n (%)									
Medicaid	23993 (37.491)	751 (18.789)	23242 (38.737)	12113 (19.190)	321 (10.282)	11792 (19.653)	24015 (38.441)	414 (16.748)	23601 (39.335)
Medicare	22281 (34.816)	2679 (67.025)	19602 (32.670)	22682 (35.934)	2204 (70.596)	20478 (34.130)	21077 (33.738)	1682 (68.042)	19395 (32.325)
Other	1698 (2.653)	62 (1.551)	1636 (2.727)	3899 (6.177)	119 (3.812)	3780 (6.300)	1635 (2.617)	38 (1.537)	1597 (2.662)
Private	13446 (21.010)	471 (11.784)	12975 (21.625)	14263 (22.596)	370 (11.851)	13893 (23.155)	12250 (19.609)	315 (12.743)	11935 (19.892)
Self	2579 (4.030)	34 (0.851)	2545 (4.242)	10165 (16.104)	108 (3.459)	10057 (16.762)	3495 (5.595)	23 (0.930)	3472 (5.787)
Race, n (%)									
Asian	3504 (5.475)	323 (8.081)	3181 (5.302)	305 (0.483)	20 (0.641)	285 (0.475)	1502 (2.404)	56 (2.265)	1446 (2.410)
Black	8041 (12.565)	482 (12.059)	7559 (12.598)	15202 (24.084)	511 (16.368)	14691 (24.485)	15624 (25.010)	443 (17.921)	15181 (25.302)
Hispanic	20459 (31.969)	864 (21.616)	19595 (32.658)	11237 (17.802)	378 (12.108)	10859 (18.098)	9751 (15.609)	239 (9.668)	9512 (15.853)
Native	341 (0.533)	18 (0.450)	323 (0.538)	64 (0.101)	1 (0.032)	63 (0.105)	175 (0.280)	5 (0.202)	170 (0.283)
Other	2953 (4.614)	174 (4.353)	2779 (4.632)	1188 (1.882)	58 (1.858)	1130 (1.883)	6291 (10.070)	234 (9.466)	6057 (10.095)
White	28699 (44.844)	2136 (53.440)	26563 (44.272)	35126 (55.648)	2154 (68.994)	32972 (54.953)	29129 (46.627)	1495 (60.477)	27634 (46.057)
Sex, n (%)									
Female	37399 (58.439)	2017 (50.463)	35382 (58.970)	38343 (60.744)	1619 (51.858)	36724 (61.207)	36928 (59.111)	1245 (50.364)	35683 (59.472)
Male	26598 (41.561)	1980 (49.537)	24618 (41.030)	24779 (39.256)	1503 (48.142)	23276 (38.793)	25544 (40.889)	1227 (49.636)	24317 (40.528)

#### Prediction of malnutrition

**Fig 1:**
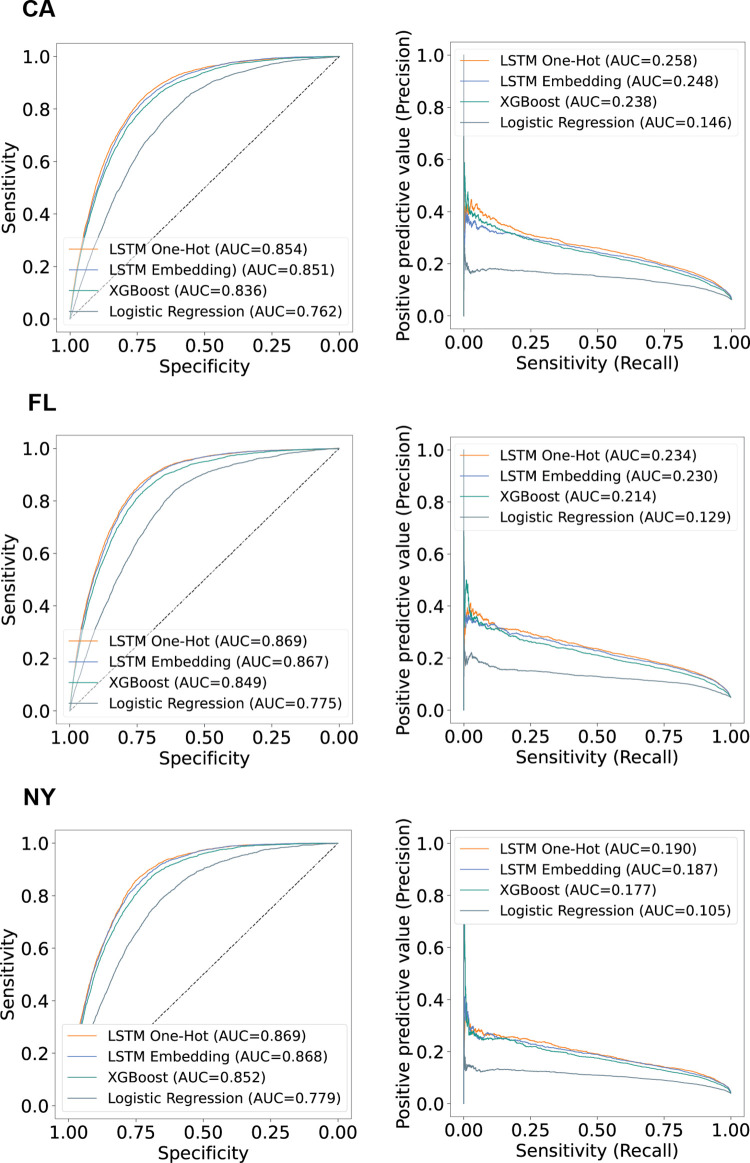
Receiver-operating characteristic (left) and precision-recall curves (right) for malnutrition prediction models, in three states. The long short-term memory (LSTM) models used longitudinal patient trajectories. The gradient-boosted tree model (XGBoost) used collapsed features from all visits. The logistic regression baseline model used only demographic data.

The best-performing model for the prediction of malnutrition was the longitudinal LSTM using one-hot encoding of diagnosis and procedure codes (Fig 1). This model, trained on patient trajectories from CA and FL, and tested on distinct cohorts from CA and FL and a completely independent dataset from NY, achieved uniformly strong predictive performance on independent test cohorts, with AUROCs ranging from 0.854 (CA, 95% CI 0.851–0.857) to 0.869 (FL, NY, 95% CI 0.866–0.872), and AUPRCs ranging from 0.190 (NY, 95% CI 0.187–0.193) to 0.258 (CA, 95% CI 0.255–0.261). Model performance is summarized in S3 Table, with bootstrap validation of CIs described in S4 Table. The best-performing embeddings (length 256, S5 Table) were slightly less performant by AUPRC, compared to the one-hot encoding strategy.

Models using longitudinal trajectories performed substantially better than models using only data from the patient’s last visit. Of the cross-sectional models, the best-performing model was a fully connected neural network, achieving AUROCs from 0.831 (CA, 95% CI 0.828–0.834) to 0.843 (FL, 95% CI 0.840–0.846), and AUPRCs from 0.162 (NY, 95% CI 0.159–0.165) to 0.233 (CA, 95% CI 0.230–0.236).

A hybrid approach, using XGBoost and collapsing features from all visits, achieved performance between that of the cross-sectional and longitudinal models: AUROCs from 0.836 (CA, 95% CI 0.833–0.839) to 0.852 (NY, 95% CI 0.849–0.855), and AUPRCs from 0.177 (NY, 95% CI 0.174–0.180) to 0.238 (CA, 95% CI 0.235–0.241).

### Patient-level risk factors

**Fig 2:**
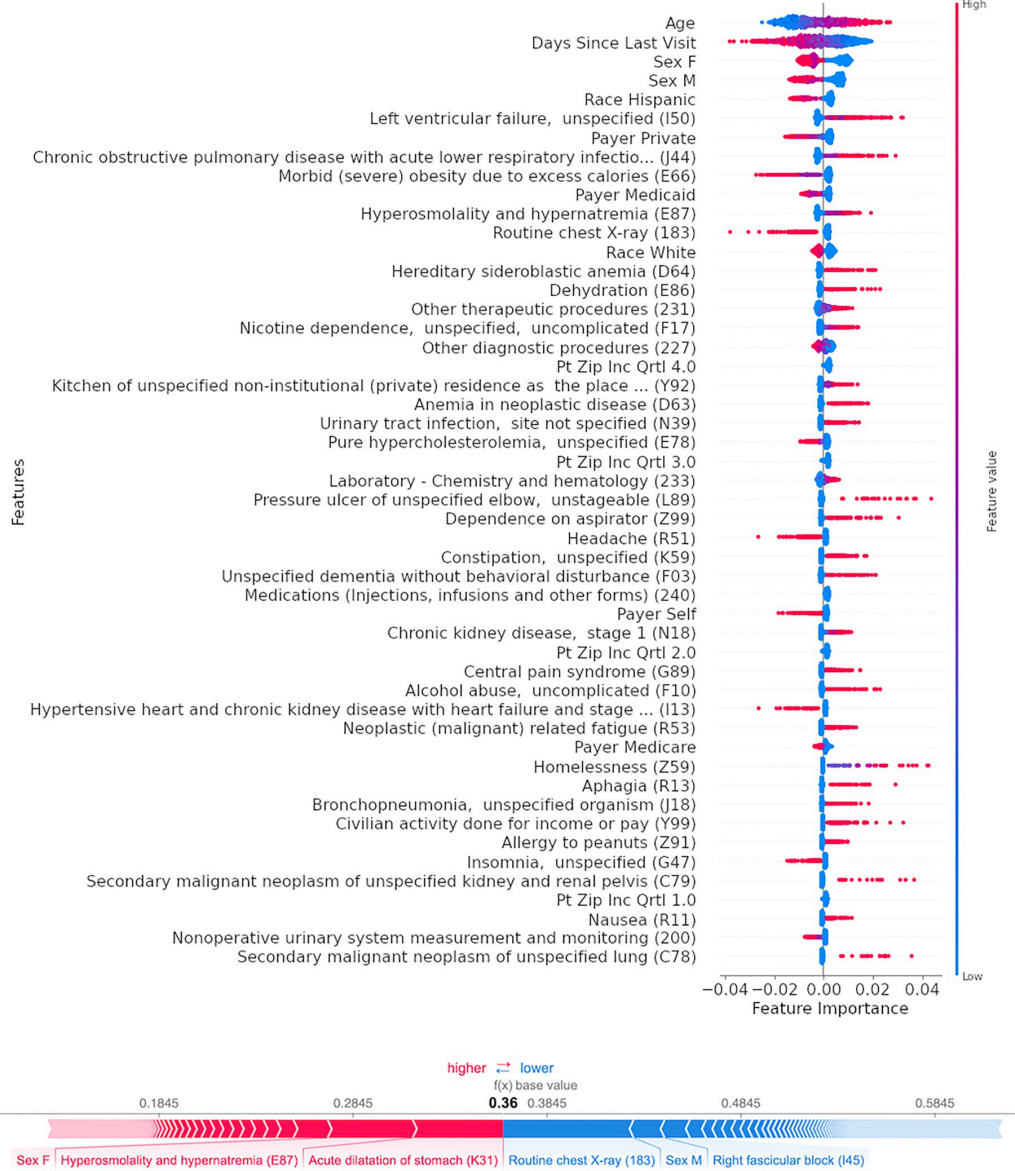
Overall and patient-specific features predictive of malnutrition. Upper panel shows the top 50 features predictive of malnutrition from the best performing model (LSTM with one-hot encoded trajectories) in descending order of influence (mean Shapley values over five visits for 1000 patients in California). Bottom panel shows risk factors for an individual malnourished patient.

The implementation of Shapley values allowed determination of patient-level and aggregate predictors of malnutrition (Fig 2). Major predictors of malnutrition identified by the model included advanced age, indicators of poor overall health (heart failure, COPD, malignancies), as well as conditions limiting nutritional intake (aphagia, food allergy, homelessness).

### Stratified analyses

In stratified analyses (S6 Table), predictive performance reflected the size and the baseline rates of malnutrition in the population of interest. For instance, models had better PPV (reflected in AUPRC) in elderly patients compared to non-elderly patients, because PPV is dependent on base rates of malnutrition, which are higher in the elderly.

### Ablation studies

When ablating categories of features to assess their relative impacts on model performance, diagnosis codes had the greatest collective effect. This was most prominent in CA, where the AUROC decreased from 0.854 (95% CI, 0.851–0.857) to 0.806 (95% CI, 0.803–0.809) with ablation of diagnostic information. Next most important were demographic data, then CCS procedure categories. Models exposed to procedure codes alone achieved an AUROC of 0.717 (95% CI, 0.714–0.720) in CA, lower than the baseline demographic models (S7 Table).

### Effect of prediction lead time

**Fig 3:**
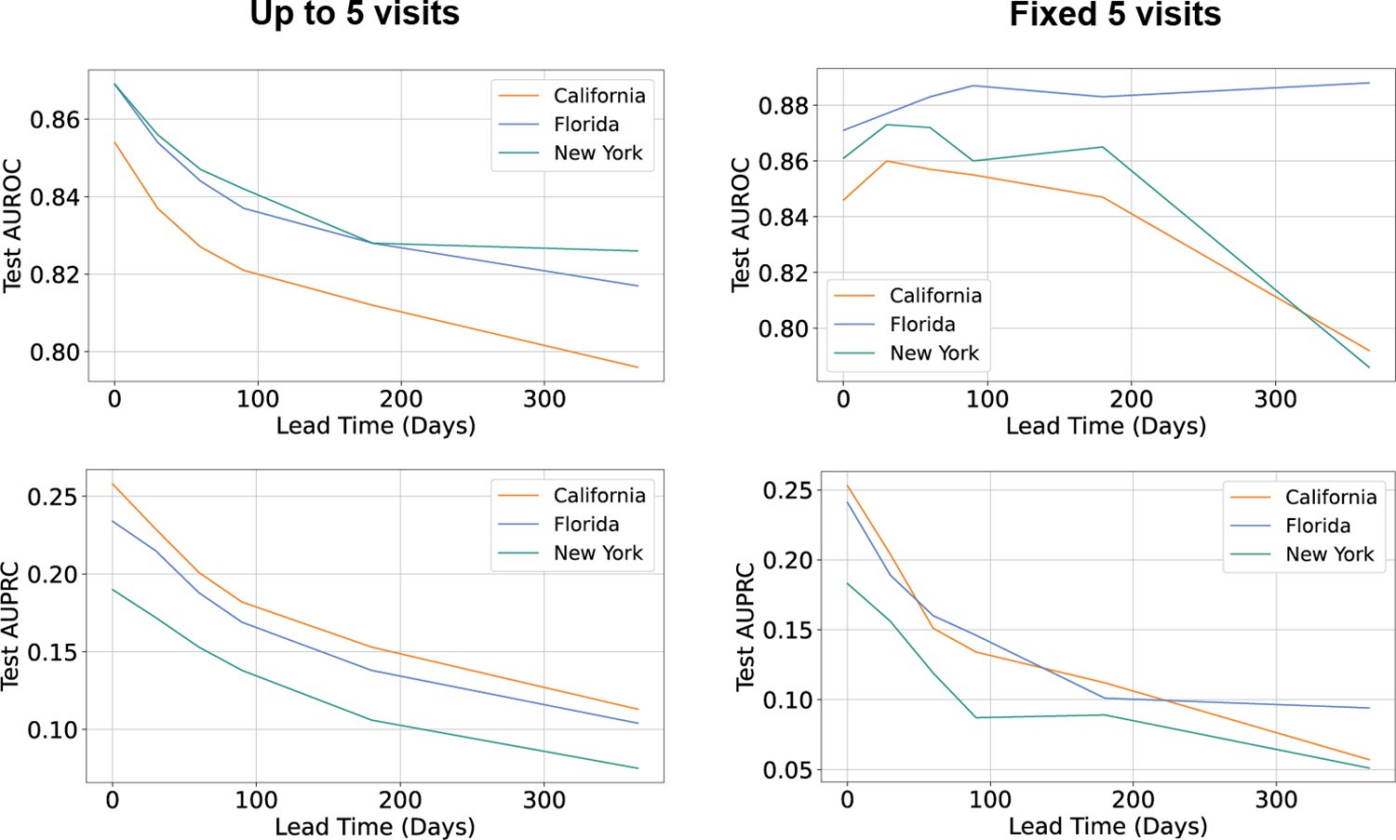
Effect of lead time on malnutrition prediction. Prediction performance of the best-performing model declines with longer intervals between prediction and diagnosis (left). Holding trajectory length constant, the detrimental effect of lead time is attenuated for AUROC but not for AUPRC (right).

Predictive performance declined with increasing lead time between the date of prediction (i.e., the last visit in each trajectory seen by the model) and the first diagnosis of malnutrition. Fewer visits are used for prediction as lead time increases, with up to 49% of the data in NY trajectories removed at a lead time of one year. Controlling for total number of visits, greater lead time remains associated with less accurate prediction (Fig 3). The steepest decline is seen between <1 month and 1–2 months, where, for instance, the AUPRC in CA drops from 0.258 (95% CI, 0.255–0.261) to 0.229 (95% CI, 0.226–0.232), though the corresponding AUROC dropped only from 0.854 (95% CI, 0.851–0.857) to 0.837 (95% CI, 0.834–0.840), suggesting a greater impact of lead time in lower-prevalence settings. AUPRC (reflective of positive predictive value) at over one year was 0.113 in CA (95% CI, 0.110–0.116), 0.104 in FL (95% CI, 0.101–0.107), and 0.075 in NY (95% CI, 0.072–0.078) (S8 Table).

## Discussion

Malnutrition is a major global comorbidity, with deleterious effects on patient trajectories and healthcare costs. Evidence-based interventions can mitigate these effects [2], but malnutrition is often overlooked until its complications are already manifest, and more difficult to correct [30]. Moreover, non-standard screening practices can produce large variation in diagnosis rates [31]. This study presents a deep learning approach to predicting malnutrition risk, using longitudinal modeling of ubiquitously recorded visit features (demographics, diagnoses, procedures). The best-performing models, which exploit the longitudinal structure of patient data, are highly accurate in predicting which patients will receive a diagnosis of malnutrition, and generalize extremely well to a population not used at all in model development. In many cases, predictions can be made with considerable lead time that could allow for focused evaluation and intervention.

The most comparable published model, MUST-Plus, is a random forest classifier trained on a single-center population used for both model development and validation, and achieved an AUROC of 0.835 [14]. In comparison, this study’s longitudinal deep learning model achieves higher performance by AUROC in all three states (0.854–0.869), and demonstrates excellent generalizability to a large, diverse population not used in model development. Moreover, whereas existing models rely heavily on laboratory or anthropometric values requiring active collection at the point of screening [14,20], this study’s approach derives most of its predictive ability from diagnostic trajectories that are passively and widely recorded, requiring no additional data collection for first-pass screening at the point of care.

While predictions were generally more accurate with shorter lead times between prediction and diagnosis (Fig 3), malnutrition diagnoses can still be predicted at clinically relevant levels even one year in advance. This provides ample opportunity to intervene on high-risk patients, which has clinical, operational, and economic benefits [32]. Somanchi et al., for instance, found that early nutritional intervention reduced average length of stay by 1.93 days, producing an annual savings of $1.16 million for 1,275 patients [33].

**Fig 4:**
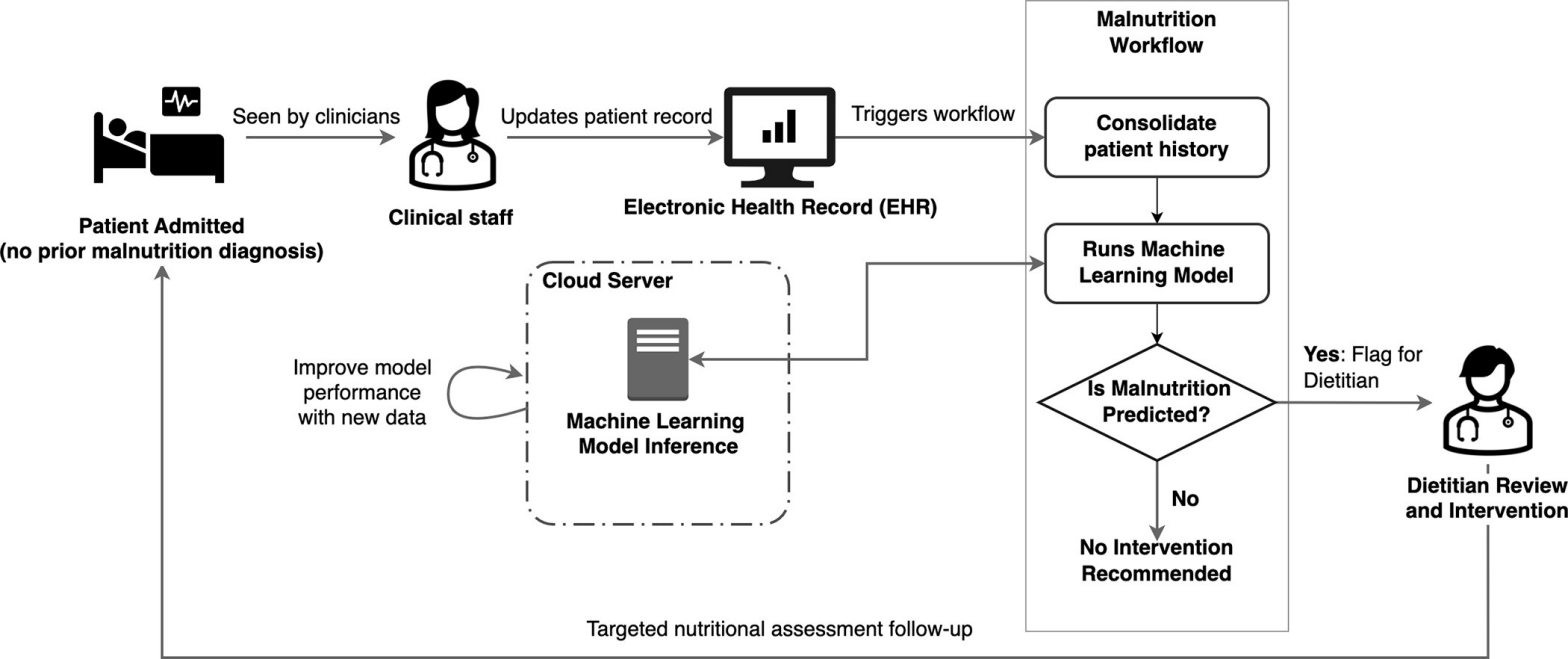
Proposed clinical implementation strategy to identify patients at risk for malnutrition and trigger dietitian referral or consultation.

Fig 4 illustrates how a model like those presented in this study could be applied in practice: upon a patient encounter (whether to clinic, ED, or hospital), the model is applied as a universal screen and flags high-risk patients without an existing malnutrition diagnosis for clinical review, using pre-existing demographic, diagnostic, and procedural data from the patient’s electronic health record. Personalized patient-level risk factors as shown in Fig 2 provide at-a-glance model interpretation that can evolve over time as additional patient visits are recorded. The provider can then assess the patient and determine whether the patient would benefit from consultation with or referral to a registered dietitian, who can then perform a targeted nutritional assessment, and suggest interventions tailored to the patient’s specific needs and risks. In current practice, malnutrition is seldom diagnosed in the ED [34], despite its being a more common point of contact with the healthcare system than hospital admissions. Automating screening for malnutrition in the ED, where providers are otherwise preoccupied with more acute diagnoses, could expose a larger and more diverse population to nutritional screening and concomitant interventions.

This study has several limitations. Because this study relied on large administrative datasets and recorded diagnoses for identification of malnutrition, it was difficult to discern whether a patient identified by the model as high-risk had yet to develop malnutrition, or was already malnourished but had not yet received a diagnosis. For many practical purposes, however, the distinction may not be important, as malnourished patients without diagnoses of malnutrition are less likely to have received focused nutritional evaluation and intervention [35], so predicting either future or undiagnosed malnutrition may be of comparable clinical benefit. Though malnutrition includes inflammatory and hypermetabolic conditions in addition to deficient intake and malabsorption, these former are less commonly diagnosed, and are poorly characterized in the data. By considering only patient trajectories with at least three visits, patients with no or minimal prior medical history were excluded, which may bias the analyses towards sicker populations at higher risk for malnutrition, though the proportions of malnourished patients in this study’s cohorts are still less than the 15–60% of hospitalized patients elsewhere estimated to be malnourished [3].

For similar reasons, relying on recorded diagnoses subjects the models to any biases in recording of diagnoses. For instance, if a group of patients had fewer diagnoses for a given rate of true malnutrition due to poorer access to healthcare, a model trained on this data could recapitulate this bias [36]. Indeed, the higher malnutrition rates in patients from wealthier communities could reflect such a bias, whereby patients from these communities are exposed to superior healthcare resources with greater capacity to diagnose and treat malnutrition. However, the limited reliance of the models on demographic compared to diagnostic features, and the excellent generalization of the models to independent test sets of different patient compositions, collectively suggest that this algorithmic screening approach is likelier to reduce than to exacerbate biases in the diagnosis of malnutrition.

## Conclusions

This study suggests opportunities for early intervention in malnutrition via automated screening of longitudinal patient records with deep learning. The best-performing model outperformed existing instruments, and generalized well to an external validation cohort. Using only passively collected demographic, diagnostic, and procedural data, this model could be easily integrated into existing clinical systems. Despite external validation in retrospective data, the model presented in this study requires prospective clinical validation, and the development of a resource-efficient mechanism for dietitian consultation or referral, before consideration for clinical use.

## Supporting information

S1 Fig Cohort creation. ED visits and admissions are grouped by patient to form trajectories. A subset of the patient trajectories is randomly sampled to create training, validation, and test sets. The training and validation datasets are derived from Florida and California data, while New York is reserved as a fully independent test set. No patients in any of the test sets are used in model development.
(PDF)

S2 Fig One-hot encoding representation of visits. Patient trajectories are pre-processed into one-hot (binary) encoded representations.
(PDF)

S3 Fig Diagnosis and procedure code embeddings. Pre-trained embeddings are created by training a two-layer fully connected network using a dataset created from target-context pairs of diagnostic and procedural codes from each visit.
(PDF)

S4 Fig Dense embedding representation of visits. Patient trajectories are transformed into dense embeddings by averaging the pre-trained code embeddings within each visit.
(PDF)

S1 Table ICD-10 diagnosis codes used as criteria for malnutrition. (PDF)

S2 Table Patient characteristics in full (non-sampled) datasets. (PDF)

S3 Table Comparison of model performance, by AUROC and AUPRC. (PDF)

S4 Table Comparison of analytical and bootstrap techniques for production of confidence intervals. (PDF)

S5 Table Effect of embedding length on prediction performance. (PDF)

S6 Table Prediction performance stratified by demographic characteristics. (PDF)

S7 Table Prediction performance in feature ablation studies. (PDF)

S8 Table Effect of lead time between prediction and diagnosis on prediction performance. (PDF)

S1 Reference (PDF)

S1 Methods Pre-trained embeddings. (PDF)
