# Supplementary material for: Predicting malnutrition from longitudinal patient trajectories with deep learning
Source: PLoS One. 2022 Jul 28;17(7):e0271487. doi: 10.1371/journal.pone.0271487 (PMC9333236; doi:10.1371/journal.pone.0271487)
Supplement: S1 Fig — ED visits and admissions are grouped by patient to form trajectories. A subset of the patient trajectories is randomly sampled to create training, validation, and test sets. The training and validation datasets are derived from Florida and California data, while New York is reserved as a fully independent test set. No patients in any of the test sets are used in model development. (PDF) [file pone.0271487.s001.pdf]

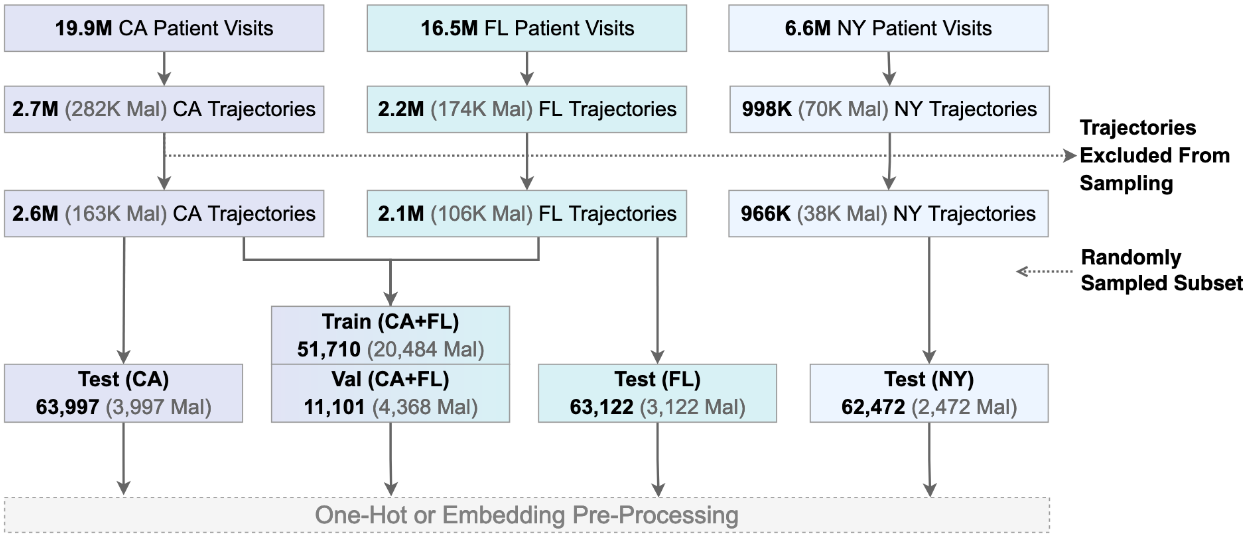

S1 Fig. Cohort creation. ED visits and admissions are grouped by patient to form trajectories. A subset of the patient trajectories is randomly sampled to create training, validation, and test sets. The training and validation datasets are derived from Florida and California data, while New York is reserved as a fully independent test set. No patients in any of the test sets are used in model development.
