# Supplementary material for: Predicting malnutrition from longitudinal patient trajectories with deep learning
Source: PLoS One. 2022 Jul 28;17(7):e0271487. doi: 10.1371/journal.pone.0271487 (PMC9333236; doi:10.1371/journal.pone.0271487)
Supplement: S2 Fig — Patient trajectories are pre-processed into one-hot (binary) encoded representations. (PDF) [file pone.0271487.s002.pdf]

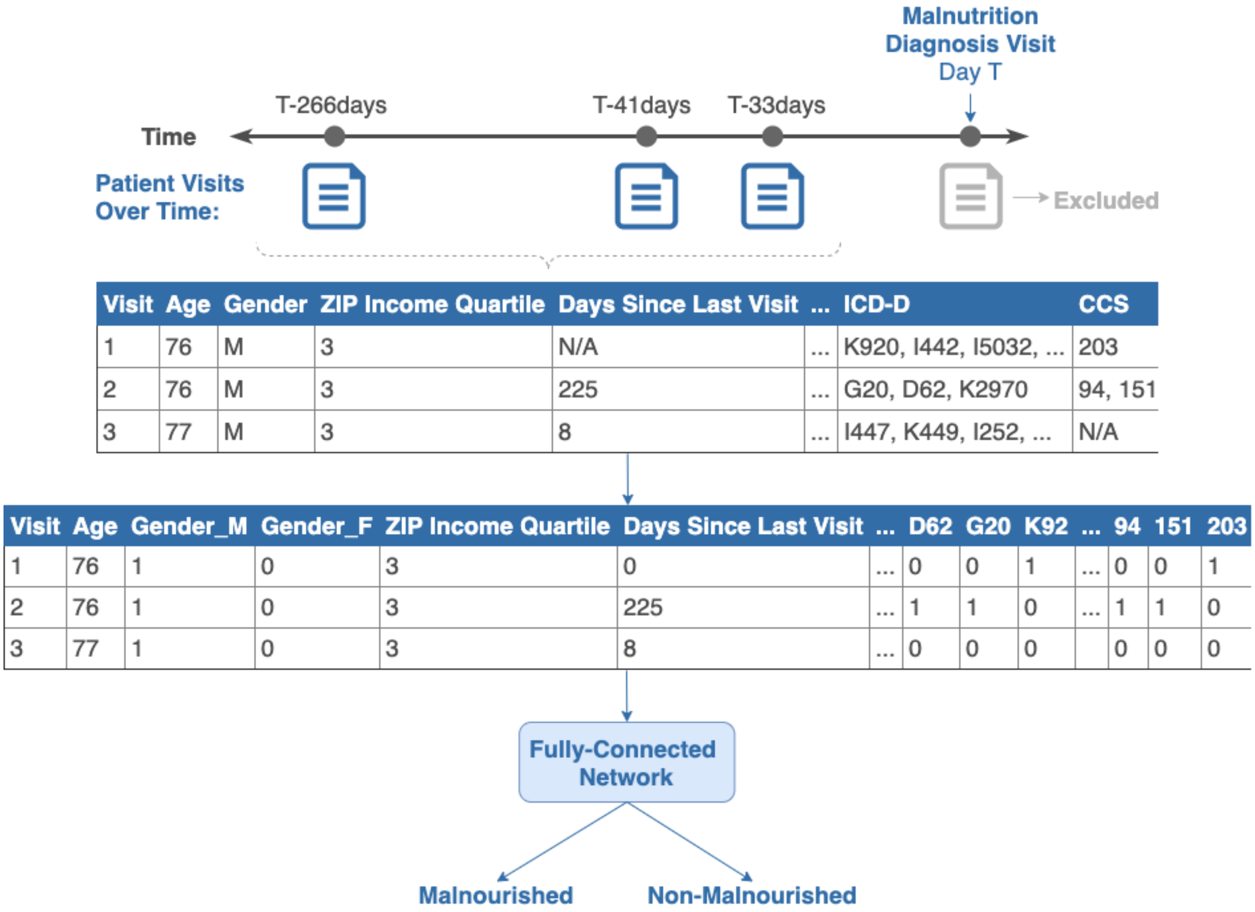


| Visit | Age | Gender | ZIP Income Quartile | Days Since Last Visit | ... | ICD-D | CCS |
|---|---|---|---|---|---|---|---|
| 1 | 76 | M | 3 | N/A | ... | K920, I442, I5032, ... | 203 |
| 2 | 76 | M | 3 | 225 | ... | G20, D62, K2970 | 94, 151 |
| 3 | 77 | M | 3 | 8 | ... | I447, K449, I252, ... | N/A |

| Visit | Age | Gender_M | Gender_F | ZIP Income Quartile | Days Since Last Visit | ... | D62 | G20 | K92 | ... | 94 | 151 | 203 |
|---|---|---|---|---|---|---|---|---|---|---|---|---|---|
| 1 | 76 | 1 | 0 | 3 | 0 | ... | 0 | 0 | 1 | ... | 0 | 0 | 1 |
| 2 | 76 | 1 | 0 | 3 | 225 | ... | 1 | 1 | 0 | ... | 1 | 1 | 0 |
| 3 | 77 | 1 | 0 | 3 | 8 | ... | 0 | 0 | 0 | | 0 | 0 | 0 |

**S2 Fig. One-hot encoding representation of visits.** Patient trajectories are pre-processed into one-hot (binary) encoded representations.
