# Supplementary material for: Predicting malnutrition from longitudinal patient trajectories with deep learning
Source: PLoS One. 2022 Jul 28;17(7):e0271487. doi: 10.1371/journal.pone.0271487 (PMC9333236; doi:10.1371/journal.pone.0271487)
Supplement: S3 Fig — Pre-trained embeddings are created by training a two-layer fully connected network using a dataset created from target-context pairs of diagnostic and procedural codes from each visit. (PDF) [file pone.0271487.s003.pdf]

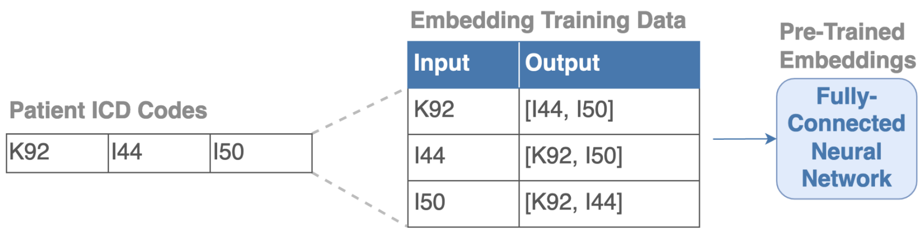

S3 Fig. Diagnosis and procedure code embeddings. Pre-trained embeddings are created by training a two-layer fully connected network using a dataset created from target-context pairs of diagnostic and procedural codes from each visit.
