# Supplementary material for: Predicting malnutrition from longitudinal patient trajectories with deep learning
Source: PLoS One. 2022 Jul 28;17(7):e0271487. doi: 10.1371/journal.pone.0271487 (PMC9333236; doi:10.1371/journal.pone.0271487)
Supplement: S4 Fig — Patient trajectories are transformed into dense embeddings by averaging the pre-trained code embeddings within each visit. (PDF) [file pone.0271487.s004.pdf]

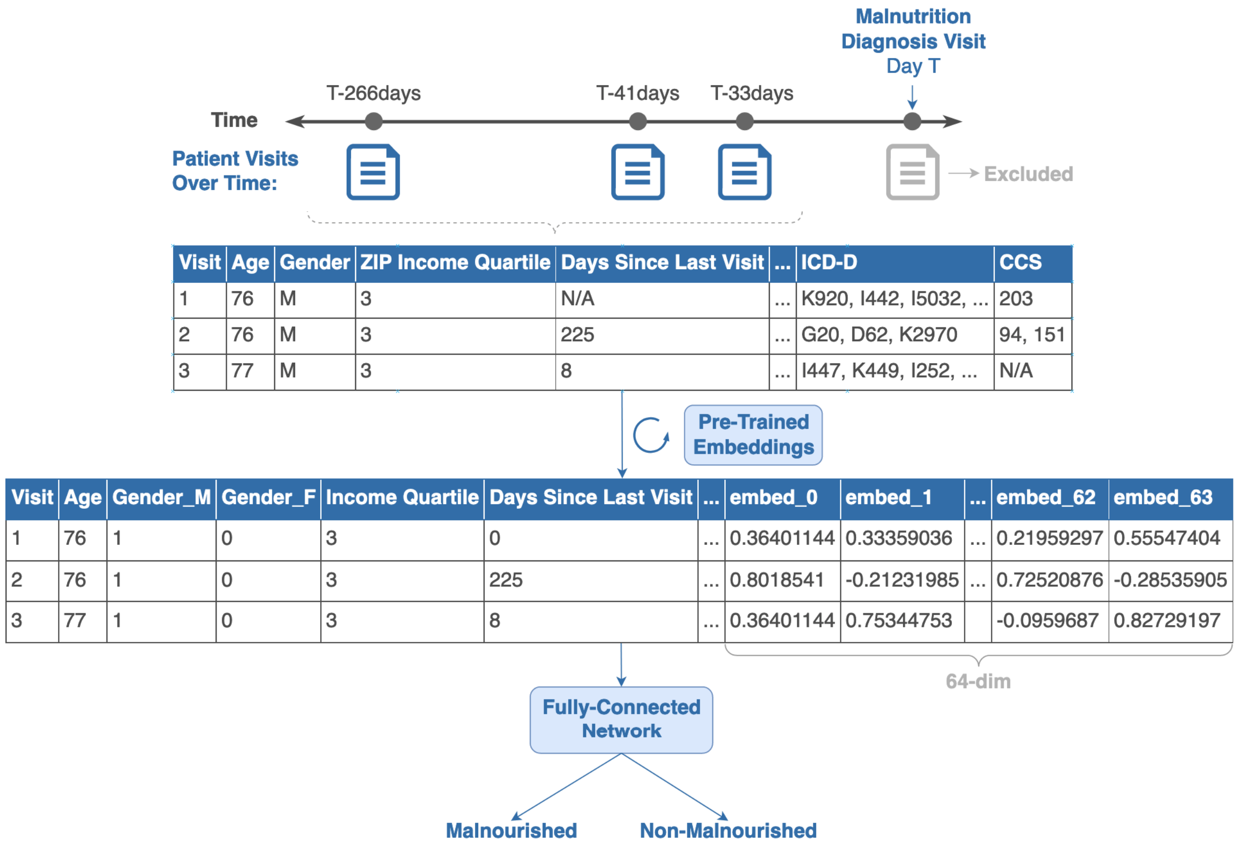

Visit	Age	Gender	ZIP Income Quartile	Days Since Last Visit	...	ICD-D	CCS
1	76	M	3	N/A	...	K920, I442, I5032, ...	203
2	76	M	3	225	...	G20, D62, K2970	94, 151
3	77	M	3	8	...	I447, K449, I252, ...	N/A

Visit	Age	Gender_M	Gender_F	Income Quartile	Days Since Last Visit	...	embed_0	embed_1	...	embed_62	embed_63
1	76	1	0	3	0	...	0.36401144	0.33359036	...	0.21959297	0.55547404
2	76	1	0	3	225	...	0.8018541	-0.21231985	...	0.72520876	-0.28535905
3	77	1	0	3	8	...	0.36401144	0.75344753		-0.0959687	0.82729197

S4 Fig. Dense embedding representation of visits. Patient trajectories are transformed into dense embeddings by averaging the pre-trained code embeddings within each visit.
