# Supplementary material for: Predicting malnutrition from longitudinal patient trajectories with deep learning
Source: PLoS One. 2022 Jul 28;17(7):e0271487. doi: 10.1371/journal.pone.0271487 (PMC9333236; doi:10.1371/journal.pone.0271487)
Supplement: S1 Table — (PDF) [file pone.0271487.s005.pdf]

S1 Table. ICD-10 diagnosis codes used as criteria for malnutrition.

ICD-10 Code	Description
E40	Kwashiorkor
E41	Nutritional marasmus
E42	Marasmic kwashiorkor
E43	Unspecified severe protein-calorie malnutrition
E440	Moderate protein-calorie malnutrition
E441	Mild protein-calorie malnutrition
E45	Retarded development following protein-calorie malnutrition
E46	Unspecified protein-calorie malnutrition
R64	Cachexia

Abbreviation: ICD-10 = 10th International Statistical Classification of Diseases and Related Health Problems.
