# Supplementary material for: Predicting malnutrition from longitudinal patient trajectories with deep learning
Source: PLoS One. 2022 Jul 28;17(7):e0271487. doi: 10.1371/journal.pone.0271487 (PMC9333236; doi:10.1371/journal.pone.0271487)
Supplement: S2 Table — (PDF) [file pone.0271487.s006.pdf]

S2 Table. Patient characteristics in full (non-sampled) datasets.

	California			Florida			New York		
	**Total**	**Malnourished**	**Control**	**Total**	**Malnourished**	**Control**	**Total**	**Malnourished**	**Control**
Sample size	2,605,485	162,724	2,442,761	2,138,959	105,792	2,033,167	965,523	38,202	927,321
Length of stay, median (IQR), days	3 (2-6)	6 (3-11)	3 (2-5)	3 (2-6)	7 (4-13)	3 (2-5)	4 (2-8)	8 (4-15)	4 (2-7)
Age, median (IQR), years	51 (33-69)	70 (58-82)	50 (32-67)	50 (32-69)	71 (58-82)	48 (32- 68)	49 (32-67)	71 (59-82)	48 (31-66)
Time from last visit, median (IQR), days	71 (15-200)	24 (7-75)	77 (16-208)	72 (17-196)	27 (10-77)	76 (18- 201)	55 (14-146)	28 (11- 69)	57 (14-149)
Income, n (%)									
Unknown	101944 (3.913)	7259 (4.461)	94685 (3.876)	75942 (3.550)	3824 (3.615)	72118 (3.547)	14143 (1.465)	662 (1.733)	13481 (1.454)
Quartile 1	548147 (21.038)	32598 (20.033)	515549 (21.105)	594236 (27.782)	26341 (24.899)	567895 (27.932)	410077 (42.472)	12345 (32.315)	397732 (42.890)
Quartile 2	763075 (29.287)	43192 (26.543)	719883 (29.470)	516842 (24.163)	24719 (23.366)	492123 (24.205)	162902 (16.872)	6007 (15.724)	156895 (16.919)
Quartile 3	690171 (26.489)	43885 (26.969)	646286 (26.457)	594618 (27.799)	29892 (28.255)	564726 (27.776)	177126 (18.345)	7478 (19.575)	169648 (18.294)
Quartile 4	502148 (19.273)	35790 (21.994)	466358 (19.091)	357321 (16.705)	21016 (19.865)	336305 (16.541)	201275 (20.846)	11710 (30.653)	189565 (20.442)
Payer, n (%)									
Medicaid	976168 (37.466)	32281 (19.838)	943887 (38.640)	411743 (19.250)	11163 (10.552)	400580 (19.702)	370036 (38.325)	6591 (17.253)	363445 (39.193)
Medicare	901168 (34.587)	108429 (66.634)	792739 (32.453)	767754 (35.894)	75330 (71.206)	692424 (34.056)	324852 (33.645)	26021 (68.114)	298831 (32.225)
Other	69732 (2.676)	1961 (1.205)	67771 (2.774)	131249 (6.136)	4052 (3.830)	127197 (6.256)	25312 (2.622)	391 (1.024)	24921 (2.687)
Private	553651 (21.249)	18911 (11.622)	534740 (21.891)	481934 (22.531)	11812 (11.165)	470122 (23.123)	190350 (19.715)	4918 (12.874)	185432 (19.997)
Self	104766 (4.021)	1142 (0.702)	103624 (4.242)	346279 (16.189)	3435 (3.247)	342844 (16.863)	54973 (5.694)	281 (0.736)	54692 (5.898)

	California			Florida			New York		
	**All**	**Malnourished**	**Control**	**All**	**Malnourished**	**Control**	**All**	**Malnourished**	**Control**
Race, n (%)									
Asian	146996 (5.642)	13297 (8.172)	133699 (5.473)	9989 (0.467)	700 (0.662)	9289 (0.457)	22802 (2.362)	1119 (2.929)	21683 (2.338)
Black	334038 (12.821)	19707 (12.111)	314331 (12.868)	517117 (24.176)	17403 (16.450)	499714 (24.578)	240677 (24.927)	7088 (18.554)	233589 (25.190)
Hispanic	829370 (31.832)	34916 (21.457)	794454 (32.523)	374626 (17.514)	12487 (11.803)	362139 (17.812)	152687 (15.814)	3743 (9.798)	148944 (16.062)
Native	13960 (0.536)	716 (0.440)	13244 (0.542)	2029 (0.095)	133 (0.126)	1896 (0.093)	2724 (0.282)	82 (0.215)	2642 (0.285)
Other	119156 (4.573)	6507 (3.999)	112649 (4.612)	41521 (1.941)	2124 (2.008)	39397 (1.938)	97631 (10.112)	3466 (9.073)	94165 (10.155)
White	1161965 (44.597)	87581 (53.822)	1074384 (43.982)	1193677 (55.806)	72945 (68.951)	1120732 (55.122)	449002 (46.504)	22704 (59.431)	426298 (45.971)
Sex, n (%)									
Female	1527442 (58.624)	82489 (50.693)	1444953 (59.152)	1299389 (60.749)	54916 (51.909)	1244473 (61.209)	569227 (58.955)	19489 (51.016)	549738 (59.282)
Male	1078043 (41.376)	80235 (49.307)	997808 (40.848)	839570 (39.251)	50876 (48.091)	788694 (38.791)	396296 (41.045)	18713 (48.984)	377583 (40.718)
