# Supplementary material for: Predicting malnutrition from longitudinal patient trajectories with deep learning
Source: PLoS One. 2022 Jul 28;17(7):e0271487. doi: 10.1371/journal.pone.0271487 (PMC9333236; doi:10.1371/journal.pone.0271487)
Supplement: S3 Table — (PDF) [file pone.0271487.s007.pdf]

S3 Table. Comparison of model performance, by AUROC and AUPRC.

			AUROC			AUPRC		
**Model**	**Number of visits**	**Features**	**California**	**Florida**	**New York**	**California**	**Florida**	**New York**
Baseline (Logistic)	Single	Demographic	0.762±0.003	0.775±0.003	0.779±0.003	0.146±0.003	0.129±0.003	0.105±0.002
Baseline (Random Forest)	Single	Demographic	0.762±0.003	0.780±0.003	0.777±0.003	0.150±0.003	0.133±0.003	0.103±0.002
XGBoost	Single	Demographic	0.785±0.003	0.802±0.003	0.801±0.003	0.170±0.003	0.157±0.003	0.121±0.003
XGBoost	Single	Demographic, ICD-10, CCS	0.814±0.003	0.826±0.003	0.830±0.003	0.212±0.003	0.189±0.003	0.157±0.003
XGBoost	Multiple (Combined)	Demographic, ICD-10, CCS	0.836±0.003	0.849±0.003	0.852±0.003	0.238±0.003	0.214±0.003	0.177±0.003
Fully-Connected	Single	Demographic, ICD-10, CCS	0.831±0.003	0.843±0.003	0.842±0.003	0.233±0.003	0.205±0.003	0.162±0.003
Fully-Connected-LSTM	Multiple	Demographic, ICD-10, CCS	**0.854±0.003**	**0.869±0.003**	**0.869±0.003**	**0.258±0.003**	**0.234±0.003**	**0.190±0.003**
Fully-Connected-LSTM with 256-Embedding	Multiple	Demographic, ICD-10, CCS	0.851±0.003	0.867±0.003	0.868±0.003	0.248±0.003	0.230±0.003	0.187±0.003

Abbreviations: LSTM = Long Short-Term Memory; XGBoost = eXtreme Gradient Boosting; ICD-10 = 10th International Statistical Classification of Diseases and Related Health Problems diagnostic codes; CCS = Clinical Classification Software procedure categories; AUROC = Area Under the Receiver-Operating characteristic Curve; AUPRC = Area Under the Precision-Recall Curve.

95% confidence intervals shown.

Best performance is **bolded**.
