# Supplementary material for: Predicting malnutrition from longitudinal patient trajectories with deep learning
Source: PLoS One. 2022 Jul 28;17(7):e0271487. doi: 10.1371/journal.pone.0271487 (PMC9333236; doi:10.1371/journal.pone.0271487)
Supplement: S4 Table — (PDF) [file pone.0271487.s008.pdf]

S4 Table. Comparison of analytical and bootstrap techniques for production of confidence intervals.

	AUROC									AUPRC								
	California			Florida			New York			California			Florida			New York		
**Type**	**Value**	**UB**	**LB**	**Value**	**UB**	**LB**	**Value**	**UB**	**LB**	**Value**	**UB**	**LB**	**Value**	**UB**	**LB**	**Value**	**UB**	**LB**
Analytical	0.854	0.851	0.857	0.869	0.866	0.872	0.869	0.866	0.872	0.258	0.255	0.261	0.234	0.231	0.237	0.190	0.187	0.193
Bootstrap	0.854	0.848	0.859	0.868	0.863	0.874	0.870	0.864	0.874	0.257	0.245	0.269	0.234	0.224	0.243	0.191	0.179	0.204

Abbreviations: AUROC = Area Under the Receiver-Operating characteristic Curve; AUPRC = Area Under the Precision-Recall Curve; UB = Upper Bound (95% confidence interval); LB = Lower Bound (95% confidence interval).

Bootstrap results use 100 bootstrap samples.
