# Supplementary material for: Predicting malnutrition from longitudinal patient trajectories with deep learning
Source: PLoS One. 2022 Jul 28;17(7):e0271487. doi: 10.1371/journal.pone.0271487 (PMC9333236; doi:10.1371/journal.pone.0271487)
Supplement: S5 Table — (PDF) [file pone.0271487.s009.pdf]

S5 Table. Effect of embedding length on prediction performance.

	California		Florida		New York	
Patients in test set						
Whole cohort	63997		63122		62472	
Malnourished	3997		3122		2472	
Control	60000		60000		60000	
Embedding length	**AUROC**	**AUPRC**	**AUROC**	**AUPRC**	**AUROC**	**AUPRC**
32	0.843±0.003	0.234±0.003	0.859±0.003	0.218±0.003	0.861±0.003	0.178±0.003
64	0.843±0.003	0.233±0.003	0.856±0.003	0.215±0.003	0.859±0.003	0.175±0.003
128	0.848±0.003	0.241±0.003	0.865±0.003	0.226±0.003	0.865±0.003	0.185±0.003
256	**0.851±0.003**	**0.248±0.003**	**0.867±0.003**	**0.230±0.003**	**0.868±0.003**	**0.187±0.003**

Abbreviations: AUROC = Area Under the Receiver-Operating characteristic Curve; AUPRC = Area Under the Precision-Recall Curve.

95% confidence intervals shown.

Best performance is **bolded**.
