# Supplementary material for: Predicting malnutrition from longitudinal patient trajectories with deep learning
Source: PLoS One. 2022 Jul 28;17(7):e0271487. doi: 10.1371/journal.pone.0271487 (PMC9333236; doi:10.1371/journal.pone.0271487)
Supplement: S6 Table — (PDF) [file pone.0271487.s010.pdf]

S6 Table. Prediction performance stratified by demographic characteristics.

	California					Florida					New York				
	**Total**	**Mal**	**Control**	**AUROC**	**AUPRC**	**Total**	**Mal**	**Control**	**AUROC**	**AUPRC**	**Total**	**Mal**	**Control**	**AUROC**	**AUPRC**
Age															
<65	44722	1531	43191	**0.880± 0.003**	0.240± 0.004	44314	1168	43146	**0.896± 0.003**	**0.232± 0.004**	45331	917	44414	**0.902± 0.003**	0.179± 0.004
≥65	19275	2466	16809	0.737± 0.006	**0.267± 0.006**	18808	1954	16854	0.748± 0.006	**0.232± 0.006**	17141	1555	15586	0.729± 0.007	**0.196± 0.006**
Sex															
Male	26598	1980	24618	0.838± 0.004	**0.267± 0.005**	24779	1503	23276	0.853± 0.004	**0.240± 0.005**	25544	1227	24317	0.855± 0.004	**0.200± 0.005**
Female	37399	2017	35382	**0.864± 0.003**	0.249± 0.004	38343	1619	36724	**0.878± 0.003**	0.229± 0.004	36928	1245	35683	**0.878± 0.003**	0.181± 0.004
Race															
White	28699	2136	26563	0.815± 0.004	0.243± 0.005	35126	2154	32972	0.837± 0.004	0.228± 0.005	29129	1495	27634	0.840± 0.004	0.194± 0.005
Black	8041	482	7559	0.878± 0.007	0.288± 0.010	15202	511	14691	**0.909± 0.006**	0.270± 0.010	15624	443	15181	**0.899± 0.005**	**0.195± 0.006**
Asian	3504	323	3181	0.845± 0.012	**0.305± 0.015**	305	20	285	0.901± 0.010	**0.368± 0.016**	1502	56	1446	0.883± 0.016	0.174± 0.019
Hispanic	20459	864	19595	**0.893± 0.004**	0.258± 0.006	11237	378	10859	0.897± 0.004	0.214± 0.006	9751	239	9512	0.887± 0.006	0.168± 0.007
Payer															
Medicaid	24421	733	23688	0.876± 0.004	0.216± 0.005	12783	332	12451	**0.904± 0.004**	0.213± 0.005	24158	401	23757	0.897± 0.004	0.148± 0.004
Private	13473	521	12952	0.885± 0.005	0.250± 0.007	14115	347	13768	0.903± 0.005	**0.247± 0.007**	12327	336	11991	**0.915± 0.005**	0.231± 0.007
Medicare	21907	2608	19299	0.755± 0.006	0.272± 0.006	22722	2208	20514	0.770± 0.006	0.241± 0.006	20759	1655	19104	0.755± 0.006	0.189± 0.005
Self	2545	68	2477	**0.891± 0.012**	**0.285± 0.018**	9958	134	9824	0.866± 0.013	0.127± 0.013	3581	41	3540	0.903± 0.010	**0.308± 0.015**

Abbreviations: AUROC = Area Under the Receiver-Operating characteristic Curve; AUPRC = Area Under the Precision-Recall Curve; Mal = Malnourished.
95% confidence intervals shown. Best performance is **bolded**.
