# Supplementary material for: Predicting malnutrition from longitudinal patient trajectories with deep learning
Source: PLoS One. 2022 Jul 28;17(7):e0271487. doi: 10.1371/journal.pone.0271487 (PMC9333236; doi:10.1371/journal.pone.0271487)
Supplement: S7 Table — (PDF) [file pone.0271487.s011.pdf]

S7 Table. Prediction performance in feature ablation studies.

	**California**		**Florida**		**New York**	
Patients						
Whole cohort	63997		63122		62472	
Malnourished	3997		3122		2472	
Control	60000		60000		60000	
Experiment	**AUROC**	**AUPRC**	**AUROC**	**AUPRC**	**AUROC**	**AUPRC**
No ablation	0.854±0.003	0.258±0.003	0.869±0.003	0.234±0.003	0.869±0.003	0.190±0.003
Ablate Demographic	0.845±0.003	0.248±0.003	0.860±0.003	0.225±0.003	0.864±0.003	0.187±0.003
Ablate CCS	0.851±0.003	0.252±0.003	0.867±0.003	0.232±0.003	0.869±0.003	0.190±0.003
Ablate ICD-10	0.806±0.003	0.191±0.003	0.828±0.003	0.175±0.003	0.821±0.003	0.133±0.003
Ablate Demographic, CCS	0.844±0.003	0.247±0.003	0.858±0.003	0.223±0.003	0.864±0.003	0.189±0.003
Ablate Demographic, ICD-10	0.717±0.003	0.145±0.003	0.787±0.003	0.141±0.003	0.786±0.003	0.118±0.003
Ablate ICD-10, CCS	0.783±0.003	0.165±0.003	0.797±0.003	0.149±0.003	0.788±0.003	0.112±0.002

Abbreviations: ICD-10 = 10th International Statistical Classification of Diseases and Related Health Problems diagnostic codes; CCS = Clinical Classification Software procedure categories; AUROC = Area Under the Receiver-Operating characteristic Curve; AUPRC = Area Under the Precision-Recall Curve. 95% confidence intervals shown.
