# Supplementary material for: Predicting malnutrition from longitudinal patient trajectories with deep learning
Source: PLoS One. 2022 Jul 28;17(7):e0271487. doi: 10.1371/journal.pone.0271487 (PMC9333236; doi:10.1371/journal.pone.0271487)
Supplement: S8 Table — (PDF) [file pone.0271487.s012.pdf]

S8 Table. Effect of lead time between prediction and diagnosis on prediction performance.

		California					Florida					New York				
		Patients					Patients					Patients				
Lead time	Trajectory length (visits)	Total	Mal	Control	AUROC	AUPRC	Total	Mal	Control	AUROC	AUPRC	Total	Mal	Control	AUROC	AUPRC
0+ days	3 - 5	63997	3997	60000	0.854± 0.003	0.258± 0.003	63122	3122	60000	0.869± 0.003	0.234± 0.003	62472	2472	60000	0.869± 0.003	0.190± 0.003
	5	32504	2106	30398	0.846± 0.004	0.253± 0.005	33609	1708	31901	0.871± 0.004	0.241± 0.005	27865	1136	26729	0.861± 0.004	0.183± 0.005
30+ days	1 - 5	62948	3865	59083	0.837± 0.003	0.229± 0.003	62446	3074	59372	0.854± 0.003	0.215± 0.003	61122	2419	58703	0.856± 0.003	0.172± 0.003
	5	20116	877	19239	0.860± 0.005	0.204± 0.006	21467	751	20716	0.877± 0.004	0.189± 0.005	16318	490	15828	0.873± 0.005	0.156± 0.006
60+ days	1 - 5	61508	3608	57900	0.827± 0.003	0.201± 0.003	61183	2892	58291	0.844± 0.003	0.188± 0.003	59199	2253	56946	0.847± 0.003	0.153± 0.003
	5	15545	523	15022	0.857± 0.006	0.151± 0.006	16538	450	16088	0.883± 0.005	0.160± 0.006	11665	256	11409	0.872± 0.006	0.119± 0.006
90+ days	1 - 5	59873	3326	56547	0.821± 0.003	0.182± 0.003	59737	2690	57047	0.837± 0.003	0.169± 0.003	57027	2045	54982	0.842± 0.003	0.138± 0.003
	5	12466	357	12109	0.855± 0.006	0.134± 0.006	13048	305	12743	0.887± 0.005	0.146± 0.006	8546	152	8394	0.860± 0.007	0.087± 0.006
180+ days	1 - 5	54738	2625	52113	0.812± 0.003	0.153± 0.003	54790	2086	52704	0.828± 0.003	0.138± 0.003	49767	1468	48299	0.828± 0.003	0.106± 0.003
	5	6593	166	6427	0.847± 0.009	0.112± 0.008	6908	108	6800	0.883± 0.008	0.101± 0.007	3578	54	3524	0.865± 0.009	0.089± 0.009
365+ days	1 - 5	43024	1592	41432	0.796± 0.004	0.113± 0.003	42686	1233	41453	0.817± 0.004	0.104± 0.003	31927	660	31267	0.826± 0.004	0.075± 0.003
	5	1908	38	1870	0.792± 0.018	0.057± 0.010	1893	29	1864	0.888± 0.014	0.094± 0.013	577	3	574	0.786± 0.033	0.051± 0.018

Abbreviations: AUROC = Area Under the Receiver-Operating characteristic Curve; AUPRC = Area Under the Precision-Recall Curve; Mal = Malnourished. 95% confidence intervals shown.
