# Supplementary material for: Predicting malnutrition from longitudinal patient trajectories with deep learning
Source: PLoS One. 2022 Jul 28;17(7):e0271487. doi: 10.1371/journal.pone.0271487 (PMC9333236; doi:10.1371/journal.pone.0271487)
Supplement: S1 Methods — (PDF) [file pone.0271487.s014.pdf]

**S1 Methods. Pre-trained embeddings.**

Dense pre-trained embeddings were developed to represent both diagnostic and procedural codes, as an alternative to the sparse one-hot encoding strategy. For each patient visit in the training set, all possible target-context pairs of diagnostic and procedural codes in that visit were considered (S1D Figure). A two-layer feed-forward neural network was trained on these code pairs, with the first layer representing the embeddings, and the second layer producing predictions for the likelihood of the remaining codes being coincident in a visit with an input code. This approach is analogous to embedding strategies used for natural language [1], without restrictions on token proximity in the sequence, since codes in a visit are not sequential in the manner of words in a sentence. The result is that each diagnosis or procedure code becomes associated with an "embedding vector", and that two codes' proximity in this vector space is proportional to the likelihood of two codes occurring together in a visit.

This model was trained over 50 epochs using binary cross-entropy loss and the Adam optimizer with a learning rate of 0.001. Pre-trained embeddings with lengths of 32, 64, 128, and 256 were produced. Each patient visit was then pre-processed by averaging the corresponding pre-trained embeddings of each diagnosis and procedure code in the visit. This produced a single dense embedding for each visit of length 53-277, depending on the size of the embedding used (21 non-embedded features for each visit + embedding vectors of length 32-256).
